# Magnetometric remote sensing of Earth and planetary oceans

**DOI:** 10.1098/rsta.2024.0089

**Published:** 2024-12-02

**Authors:** Robert Tyler, Hiroaki Toh, Krishan Khurana, Ikuko Fujii

**Affiliations:** ^1^ Planetary Magnetospheres Laboratory, NASA Goddard Space Flight Center, Greenbelt, MD 20771, USA; ^2^ Data Analysis Center for Geomagnetism and Space Magnetism, Graduate School of Science, Kyoto University, Kitashirakawa-Oiwake Cho, Sakyo-ku, Kyoto 606-8502, Japan; ^3^ Department of Earth Planetary and Space Sciences, University of California Los Angeles, Los Angeles, CA 90095, USA; ^4^ Meteorological College, Japan Meteorological Agency, 7-4-81 Asahi-machi, Kashiwa, Chiba 277-0852, Japan

**Keywords:** motional induction, remote sensing, ocean heat content, tsunami, ocean worlds

## 1. Introduction

Oceans in the Solar System and likely beyond have the interesting property of presenting as thin, global-scale electrical conductors sandwiched between relative insulators. Because the length scale for geometric attenuation of magnetic fields associated with electric currents in the conductor is set by the length scale of the electric currents, it is the large-scale systems that can be best observed from remote magnetic observatories including those flown on spacecraft. Because of the aspect ratio described, large-scale electric currents tend to be confined to flow in horizontal paths within the ocean.

Electric currents in the ocean can be *induced* by external sources and/or *motionally induced* by flow sources. A distinguishing feature of motional induction is that it is the flow kinetic energy that is being converted into electromagnetic energy as the flow interacts with the background magnetic field and is subject to the Lorentz force law. In the case of induction, the source of energy is external to the ocean and the energy propagates as field energy into the ocean where part of the energy and momentum is transferred to material charge carriers, resulting in electric currents.

In terrestrial studies, there is a wide body of literature treating the induced currents in the ocean and solid earth. The collection of papers in this issue is aimed at addressing the much less developed topic of motional induction due to ocean flow sources (induction papers are, however, admitted in the planetary science application). Note that in this application the large-scale flow and the large-scale electric currents are both constrained to flow predominantly in horizontal loops within the ocean. As such, one may expect distinguishing features in the types of processes that occur, as well as an opportunity for imposing specialized mathematical approximations that efficiently address this class of application.

The articles in this issue are collected into three nominal categories of applications (Physical Oceanography, Natural Hazards, Planetary Studies) which we now introduce in the following subsections.

## 2. Physical Oceanography

As described above, the development underway of magnetometric remote sensing has focussed on large-scale electric currents generated through motional induction by large-scale ocean flow sources. In studies classified here as Physical Oceanography and aimed at the opportunities that have opened in the modern era of satellite geomagnetic observatories, these flow sources are the global ocean tides and general circulation.

The studies involving the tides are the most developed in terms of validation of models with observations. Indeed, agreement has been demonstrated between forward models predicting the tidal magnetic fields and the fields extracted from satellite magnetic data such that the ocean’s lunar tidal magnetic field component is now an L2 product distributed with results from the Swarm satellite magnetic survey underway. In ‘Magnetic signals from oceanic tides: new satellite observations and applications’ by Grayver *et al*. [1], the reader will find review material on this topic as well as an example of the latest extractions of ocean tidal signals and advances in forward modelling of the ocean tidal fields.

While it has been useful to develop an understanding and models of the ocean tidal components of the geomagnetic field, a primary driver of development in this field has been the opportunities for using the ocean tidal signals as a source for sounding the ocean (and solid Earth) electrical conductivity as well as the ocean tidal flow. The most highly motivated opportunity stems from the dependence of ocean electrical conductivity on ocean temperature. Because the remotely observable tidal magnetic fields are sensitive to depth integrals of the ocean sources (a dependence shared by gravity studies but not most other ocean remote sensing methods), there is an opportunity for magnetometric remote sensing of ocean heat uptake due to the changing climate. In ‘Sensitivity of M_2_ tidal magnetic signals to seasonal and spatial variations of ocean electric conductivity’ by Velímský and Šachl [2], the sensitivity of the ocean tidal magnetic fields to seasonal variations in ocean conductivity is examined and an inverse method for constraining the upper ocean conductivity using remote magnetic data is demonstrated.

Modern satellite magnetic surveys provide unprecedented spatial coverage of the ocean tidal magnetic fields but land geomagnetic observatories continue to provide superior temporal resolution. Moreover, the long historical records from the land observatories offer information about past ocean variability. In ‘Oceanic and ionospheric tidal magnetic fields extracted from global geomagnetic observatory data’ by Tyler and Trossman [3], the tidal signals are systematically extracted from data from the global collection of land observatories and an attempt is made to distinguish the parts due to tides in the ocean versus tides in the ionosphere.

The current success in extracting the very weak magnetic signals due to tides has hinged on the high predictability of the astronomical tidal forces and the ability to average across tidal cycles. The ocean circulation also generates large-scale remote magnetic fields but the extraction of these signals from observations has been more challenging given the reduced predictability of ocean circulation and overlap of its space/time scales with other incompletely resolved magnetic field sources. But the amplitudes of the competing signals vary with frequency and spatial scale and there can be improved opportunities for extracting the oceanic signals at sub-tidal periods and/or through scale selection. A study to determine the space/time scales of the ocean circulation signals that can be best separated currently is treated in ‘Satellite monitoring of long period ocean-induced magnetic field variation’ by Finlay *et al*. [4], which also includes a useful overview of geomagnetic field modelling and the Swarm magnetic mission. In this case, the extractability of the oceanic signals is studied using forward-model simulations of the magnetic fields due to ocean flow taken from an ocean state model.

Simulated magnetic fields driven by an ocean model state are similarly used in ‘Physical oceanographic factors controlling the ocean circulation-induced magnetic field’ by Trossman *et al* [5]. The simulation results are studied to derive the oceanographic factors controlling the magnetic field variability and also to examine the augmented oceanographic budget analyses that include the electrodynamic variables and parameters. In this case, extraction of the ocean circulation magnetic signals is not directly the goal but rather the determination of the information content of the magnetic signals and how magnetic field observations might be assimilated together with other data in ocean modelling.

## 3. Natural Hazards

The Natural Hazards collection consists of three studies on tsunamigenic magnetic fields. Tsunamis are an example of large-scale ocean waves with far-reaching magnetic fields that are motionally induced by the associated flow. The momentum and energy density in the tsunami wave increases as the wave enters shallow water and the wavelength shortens, resulting in the deposition of enormous energy in a rather short time period and coastal disasters.

Electromagnetic fields generated by tsunamis have been studied both theoretically and observationally, with most of the studies appearing in the last decade or so. The tsunami-generated magnetic field arrives before the fluid wave itself. This has given the possibility of using the tsunamigenic magnetic field in tsunami warning systems.

Detecting tsunami-generated electromagnetic fields from land could be more efficient than detecting them from ocean bottom observatories, as is currently done. ‘Properties of tsunami-generated electromagnetic variation observed on islands’ by Minami [6] explores this opportunity through numerical simulation of a case assuming an idealized island.

Ocean bottom pressure gauges sensing the sea surface displacement are often used to detect tsunamis. The tsunami-generated electromagnetic fields are vectors, therefore they contain more information than that of the scalar pressure observations. ‘Estimation of tsunami direction and horizontal velocity field from tsunami magnetic field’ by Lin [7] attempts to infer characteristics of the tsunami wave from the tsunami-generated magnetic field and claims that they successfully estimate propagation information for two actual tsunami cases.

‘Harnessing electromagnetic data for tsunami source estimation: a comprehensive review’ by Baba *et al*. [8] aims further. They present a review on studies to use the tsunami-generated electromagnetic field as a tool to describe the earthquake causing the tsunami. New information from the tsunami-generated electromagnetic field to estimate a slip distribution on a fault is explored.

## 4. Planetary Studies

Most of the effort currently on understanding the satellites’ oceans is devoted towards crafting studies on future missions that can exploit the electromagnetic induction signals emanating from their rotating planets. As orbiting the satellites of Uranus and Neptune is beyond the current capabilities of our spacecraft technologies, novel techniques are required to establish the presence and properties of subsurface oceans in their satellites from a very limited number of satellite flybys. In ‘Dual-frequency electromagnetic sounding of a Triton ocean from a single flyby’, Khurana *et al*. [9] show that the extremely tilted magnetic field of Neptune and the large inclination of Triton’s orbit create two strong magnetic signals in Triton at the synodic rotation period of Neptune (14.4 h) and the orbital period of Triton (141 h). They further exploit the small phase delays of the induced fields by the putative ocean to determine the complex response of Triton at these frequencies from a single flyby. The two complex responses allow the authors to distinguish between induction responses made by the very conducting ionosphere of Triton and its ocean.

Another major target of future missions is the Uranus system with its five large satellites, Miranda, Ariel, Umbriel, Titania and Oberon. Once again, only a few multiple flybys of each of these satellites would be permissible for a future mission due to cost constraints. In, ‘On detecting and characterizing planetary oceans in the solar system using a distance-based ensemble modelling approach: application to the Uranus system’, Cochrane *et al*. [10] use a big-data modelling approach that uses the technique of principal component analysis to determine the location, size and conductivity of Umbriel’s putative ocean from just three flybys. The key to a successful inversion is the exploitation of multiple frequencies (at least two) so that all three ocean parameters can be determined with an error of <50%.

## Data Availability

This article has no additional data.
